# Management of a parotid sialocelein a young patient: case report and literature review

**DOI:** 10.1590/S1678-77572010000400019

**Published:** 2010

**Authors:** Melissa Rodrigues de ARAUJO, Bruna Stuchi CENTURION, Danielle Frota de ALBUQUERQUE, Luiz Henrique MARCHESANO, José Humberto DAMANTE

**Affiliations:** 1 DDS, MSc, PhD. Department of Stomatology, Bauru Dental School, University of São Paulo, Bauru, SP, Brazil.; 2 DDS, Department of Stomatology, Bauru Dental School, University of São Paulo, Bauru, SP, Brazil.; 3 DDS, MSc. Department of Stomatology, Bauru Dental School, University of São Paulo, Bauru, SP, Brazil.; 4 MSc, PhD. Clinical Analysis Laboratory, Craniofacial Anomalies Rehabilitation Hospital, University of São Paulo, Bauru, SP, Brazil.; 5 DDS, MSc, PhD, Full Professor. Department of Stomatology, Bauru Dental School, University of São Paulo, Bauru, SP, Brazil.

**Keywords:** Parotid gland, diagnosis, Sialography, Sialocele

## Abstract

Sialocele is a subcutaneous cavity containing saliva, caused by trauma or infection in the parotid gland parenchyma, laceration of the parotid duct or ductal stenosis with subsequent dilatation. It is characterized by an asymptomatic soft and mobile swelling on the parotid region. Imaging studies are useful and help establishing the diagnosis, such as sialography, ultrasonography, computed tomography and magnetic resonance imaging. This paper describes a recurrent case of a parotid sialocele in a young female patient. She presented a 6 cm x 5 cm swelling on the left parotid region. The ultrasonographic scan of the area revealed a hypoechoic ovoid well defined image suggesting a cyst. A sialography of the left parotid showed a cavitary sialectasia in a panoramic and anteroposterior view. A conservative management was adopted by percutaneous needle aspiration of the swelling, which was useful to provide material for analysis and helped healing. Dentists should be aware of this pathology and the importance in adopting a conservative treatment whenever it is possible.

## INTRODUCTION

Sialocele is a subcutaneous cavity containing saliva, usually results from trauma or infection to the parotid gland parenchyma, laceration of the parotid duct or ductal stenosis with subsequent dilatation^5.^ A post-traumatic sialocele is an acquired lesion that arises from extravasation of saliva into the glandular or periglandular tissues secondary to disruption of the parotid duct or parenchyma^1,7^. Traumatic causes include sharp penetrating wounds in the oral cavity or in the face^6,17^ and blunt trauma, such as zygomatic and mandible fractures^15,19^. extrinsic infections from mandibular teeth^3^ mumps, actinomycosis, tuberculosis and syphilis have been recognized as causes of parotid fistulae in the past^14^.

Congenital fistulae and the ones secondary to invasive malignant tumors of the parotid gland or the oral cavity can also be associated to sialocele^15^. Temporomandibular joint surgery^13^, parotidectomy^18^, mastoidectomy^12^, mandibular osteotomies^11^ and facial abscess drainage^25^ have been mentioned as potential causes of sialocele, and in all these cases the duct and/or the gland are damaged. Patient’s history usually includes facial trauma or surgery, days or weeks before the onset of the swelling^5^.

Sialocele is characterized by a swelling on the parotid region. On palpation, the lesion may be soft and mobile and unless secondary infected, the patient has no pain, fever, chills, or erythema of the skin^7^.

The diagnosis is complex and involves a combination of thorough history and clinical assessment of the patient, fine needle aspiration and image analysis. Fine needle aspiration is a standard technique which permits sampling and the fluid may be submitted to further investigation. Imaging studies include sialography, ultrasonography, computed tomography (CT) and magnetic resonance imaging^5,7^. The sialography is a technique that may increase sialocele’s pressure causing rupture and fistulae, although this is not a commonly observed complication^18^. CT may show details of the area, such as a single or multiloculated cystic lesion with regular margins and lower density of the surrounding tissues. The CT differential diagnosis would include retention cyst, sialodochitis, branchial cleft cyst and lymphoepithelial cyst^7^.

Many treatment modalities have been mentioned in the literature. They basically consist of a conservative or a surgical approach. A conservative modality is based on regular aspiration of the content, compression dressing, and administration of an antisialogogue^8,18^. Radiotherapy and toxins are other treatment modalities. Administration of botulinum toxin causes temporary chemical denervation of the cholinergic nerve fibers and has been used successfully. It is a highly effective, safe and non invasive method^21,22,28^. Drugs act by blocking acethylcoline release, thereby inhibiting neurotransmission at the secretomotor parasympathetic autonomic nerve ending responsible for salivation^9^. When conservative management fails, or when the overlying skin become so thin that there is imminent risk of rupture, surgical treatment is indicated^11^. Surgery may be performed by repair or reconstruction of the duct, creation of a controlled internal fistula, superficial or total parotidectomy, parasympathetic denervation, and ductal ligation. If the sialocele is left untreated, it may develop into a significantly large facial swelling, fistula formation and may drain extra orally^8,29^

This paper reports a case of a parotid sialocele in a young patient managed with a conservative approach.

## CASE REPORT

A white17-year-old female patient presented with a 4-month history of swelling over her left cheek anteriorly to the ear. She referred 3 previous episodes, the last one having started 1 month before the appointment. She denied trauma to the region, had not have episodes of fever lately and her medical history was not contributory. There was no associated pain or alteration of facial function as well as no motor or sensory deficits were observed.

**Figure 1:**
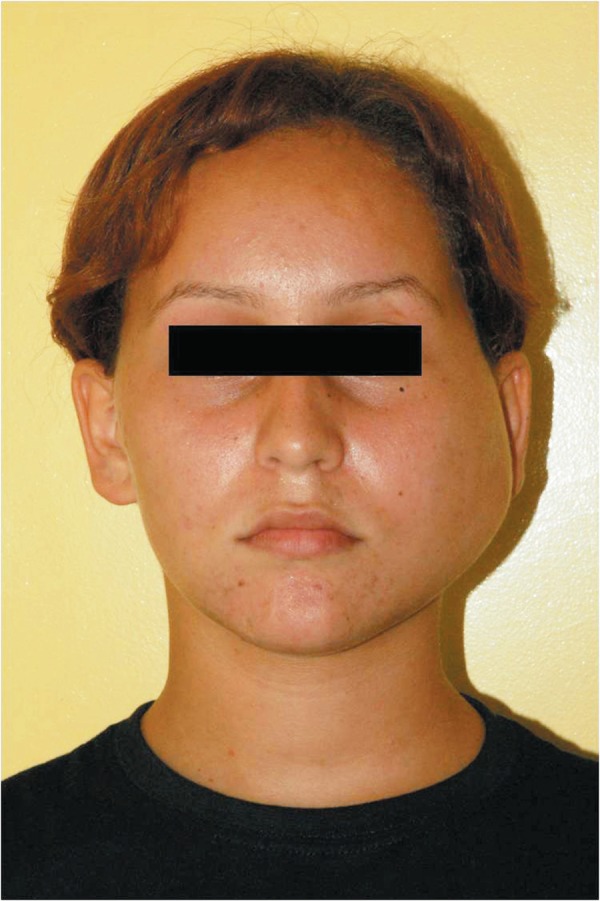
Anteroposterior facial view illustrating the 6 x 5 cm swelling on patient’s left cheek

**Figure 2:**
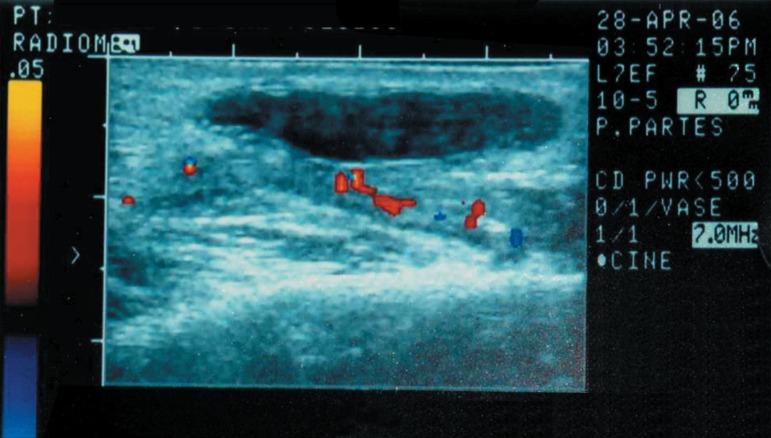
The longitudinal section in the ultrasonographic scan of the left parotid gland demonstrates a hypoechoic ovoid well-defined image suggesting a cyst

**Figure 3:**
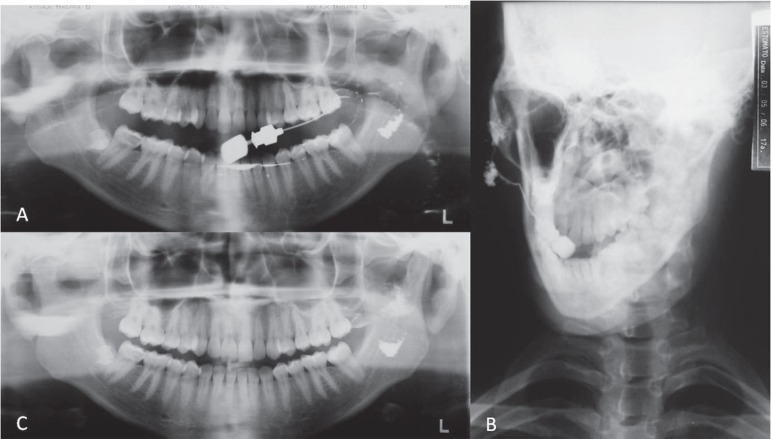
A panoramic (A) and an anteroposterior (B) radiograph taken after sialography of the left parotid gland. A partial filling of the gland was sufficient to show cavitary sialectasia. The contrast was retained in the gland for at least 24 h, as shown in the panoramic radiograph (C)

The swelling measured about 6 cm in length and 5 cm in width. On bimanual palpation an ill-defined and resilient mass was noticed (Figure 1). This mass was evident extra orally with a considerable bulging of the skin in the left parotid region. The lesion was normal and no lymphadenopathy was detected. On intraoral examination, oral mucosa and teeth were healthy. The parotid duct in the affected side was normal and salivary flow had normal physical aspects without debris or purulent discharge. The presumptive clinical diagnosis was an abscess associated to mild inflammation, considering the patient’s history and the previous episodes. Conventional panoramic radiography, ultrasonography and sialography were performed. The panoramic radiograph showed teeth and bones preserved without any evidence of abnormalities. The ultrasonographic scan revealed a hypoechoic ovoid well defined image suggesting a cyst (Figure 2). Sialography of the left parotid was performed using Lipiodol^®^ (Lipiodol; Guerbet, Jacarepaguá, RJ, Brazil) as a substance of contrast. A partial filling of the gland was enough to show cavitary sialectasia in a panoramic (Figure 3a) and anteroposterior view (Figure 3b), and the contrast was retained in the gland for at least 24 hours (Figure 3c).

**Figure 4:**
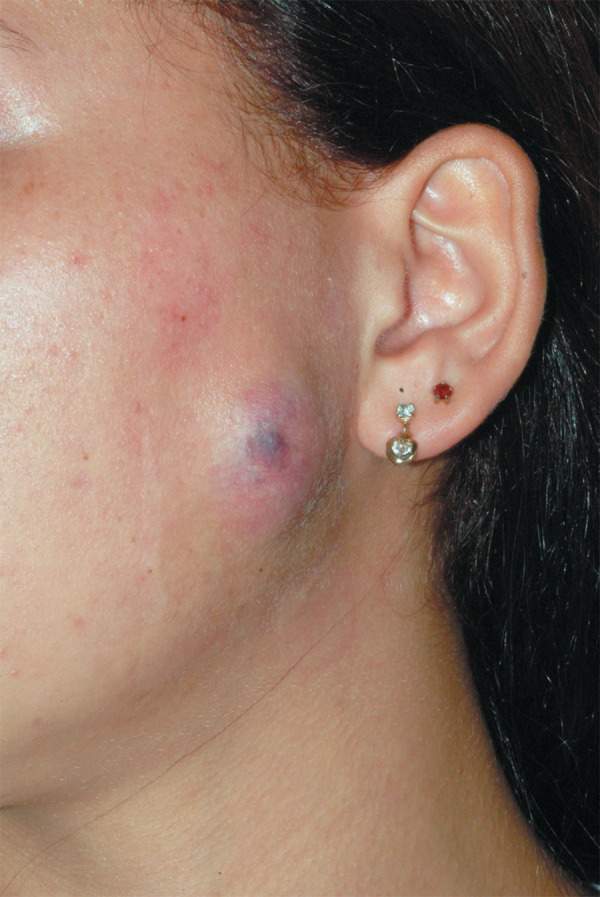
Erythema on the overlying skin after the percutaneous needle aspiration

**Figure 5:**
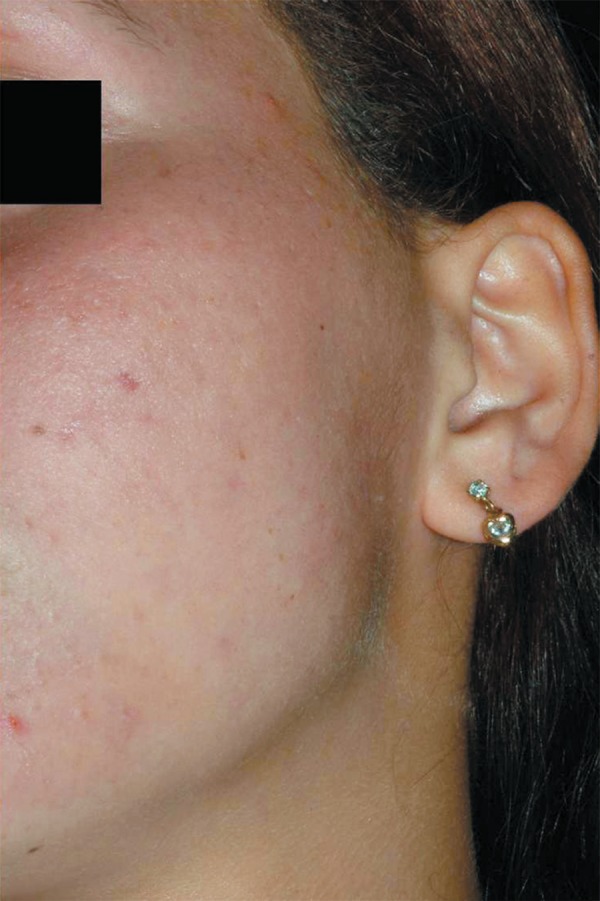
Complete recovery of the skin and remission of the swelling after 30 days

A percutaneous needle aspiration of the swelling was performed 2 weeks after the sialography. It yielded 4 mL of a clear viscous fluid. The material was submitted to microbiological analysis and showed numerous polymorphonuclear leukocytes. The fluid did not show any bacterial growth and presented high amylase levels (7,810 units/L). The aspiration procedure was sufficient to empty the cavity. The patient was advertised to compression dressing twice a day. Seven days after the percutaneous aspiration a great decrease of the swelling was observed. The overlying skin showed a discreet erythema on the surface of the mass, without signs of infection, and the patient did not refer pain (Figure 4). At the 30-day follow-up visit, complete recovery was noticed, and the residual swelling had totally disappeared (Figure 5). The patient was followed up during two years and a half and no recurrence was detected.

## DISCUSSION

Primary parotid gland cysts are very rare, representing five per cent of all parotid tumors^10,23,25^,. Mucoceles are round and well defined lesions that contain mucus, when they occur in the parotid gland are called sialoceles. For practical purposes they may be regarded as being of either extravasation or retention type. The term mucous extravasation phenomenon (cyst) is used when mucus is extruded into the connective tissues and is surrounded by a granulation tissue, while mucous retention cyst is used to describe a cyst with retained mucin that is lined with ductal epithelium which may have undergone squamous or oncocytic metaplasia^26,27^. The factors that determine a mucocele are the rate of mucus production and the speed of phagocytosis of the extravasated mucus. The majority of the mucoceles previously reported in the parotid gland are of retention type (ductal cyst)^26^.

Parotid sialoceles are lesions that occur after trauma or injury in the face causing accumulation of saliva in the area^1,4,20^. It has not been described yet a case where the patient could not associate a history of trauma, injury or surgery. It is possible that our patient suffered a trauma of low intensity and could not remember it, but it should be something else to explain the 3 recurrent episodes of tumefaction.

Initially, our patient had a resilient, ill-defined mass, which was difficult to palpate, probably due to the position of the cystic diffuse inflammation, under the dense parotid fascia, which makes physical examination unreliable. Clinical assessment may be inaccurate in these cases^4,24^. However, soft and mobile lesions had also been described when they are more superficial^7^.

Our patient did not present fever or other compromising signs in any episode of swelling^20^.

The management of a patient with a swelling in the parotid region requires careful clinical evaluation. Fine-needle aspiration or biopsy is necessary for a definite diagnosis. Sialography, computed tomography and ultrasonographic scans may be very helpful^2^. Sialography of the parotid gland was mandatory in revealing the sialectasia in our case and is also useful to distend the duct when it is involved^4^. Lipiodol^®^ is a solution containing iodine that might have acted as an antibacterial agent and helped reducing the facial swelling combined with the aspiration. In our case report the sialography served as a diagnostic tool and helped healing the involved gland. There was a clear relief of the signs after sialography.

Another key to the diagnosis and treatment was fine-needle aspiration, which provided material for analysis and helped empting the gland. Parotid secretion has a high amylase content that is usually around 10,000 units/L^19^. In the present case, the amylase content was 7,810 units/L, confirming the diagnosis of parotid saliva extravasation.

A variety of treatments have been proposed for parotid sialoceles^20^. These include multiple aspirations and compression dressings; late primary repair or reconstruction of the duct; creation of a controlled internal fistula; superficial or total parotidectomy; parasympathetic denervation (sectioning of the auriculotemporal nerve); antisialogogues; radiation therapy and ductal ligation. Most of these procedures are invasive with risks of injury of the facial nerve, with variable and often poor success rates^7^. The anticholinergic drugs have many undesired side effects such as xerostomia, constipation, photophobia, tachycardia and urinary retention^16^. Atropine and glycopyrrolate are antisialagogue drugs that may be used to treat sialoceles^4^. The present case reported did not require any invasive treatment or administration of a drug. A conservative treatment such as suggested by Landau and Stewart^17^ (1985), the injection of an antibacterial solution to perform a sialography, the aspiration of the content and the compression dressing were capable to solve the case.

Dentists should be aware of this pathology and the importance in adopting a conservative treatment whenever it is possible.
